# Supplementary material for: Antimicrobial Management of Skin and Soft Tissue Infections among Surgical Wards in South Africa: Findings and Implications
Source: Antibiotics (Basel). 2023 Jan 31;12(2):275. doi: 10.3390/antibiotics12020275 (PMC9951966; doi:10.3390/antibiotics12020275)
Supplement: Supplementary file 1 [file antibiotics-12-00275-s001.zip › antibiotics-2175667-supplementary.pdf]

Supplementary Table S1: Data collecting instrument

**Skin and soft tissue infections**			
**Patients details**			
Date:		Study no:	
Ward:	Bed:		
Admission date:			
Discharge date:		Dr:	

Gender:	Weight:	Height:

Allergy	Y	N	Smoking	Y	N	Alcohol	Y	N	Caffeine	Y	N

Vital signs upon admission:<br>BP:<br>Temp:<br>Resp:<br>Pulse:	Other chronic conditions:

**Assessing for SSTI**					
The type of SSTIs:					
Uncomplicated SSTI?	Y	N	If yes, which type of uncomplicated SSTIs?		
			Erysipelas	Impertigo	Mild cellulitis
			Edema	Other?	
Complicated SSTIs?	Y	N	If yes, which type of complicated SSTIs?		
			Severe infective cellulitis	Ulcers/ wound site infection	Surgical site infection
			Major abscesses	Infected burns	Skin ulcers
			Diabetic foot ulcers	Other?	

Clinical symptoms upon admission:	
History of SSTI in the past:	
History of surgery in the infected area:	
Abscess incision and drainage in the past year:	

Culture done?	Y	N	If yes, which pathogen was found?

**Assessing antimicrobials**			
Antimicrobial exposure in the last month?	Y	N	Reasons?

Antibiotics information	Drug 1	Drug 2	Drug 3	Drug 4
Name:				
Dose:				

Frequency:				
Route:				
Start date:				
Stop date:				
Is the treatment in line with SA guidelines?				
